# Identifying intersectional groups at risk for missing breast cancer screening: Comparing regression- and decision tree-based approaches

**DOI:** 10.1016/j.ssmph.2024.101736

**Published:** 2024-12-09

**Authors:** Núria Pedrós Barnils, Benjamin Schüz

**Affiliations:** Institute for Public Health and Nursing Research, University of Bremen, Bremen, Germany

**Keywords:** Quantitative intersectionality, Decision trees, Breast cancer screening, Health inequalities, Germany

## Abstract

Malignant neoplasm of the breast was the fifth leading cause of death among women in Germany in 2020. To improve early detection, nationwide breast cancer screening (BCS) programmes for women 50–69 have been implemented since 2005. However, Germany has not reached the European benchmark of 70% participation, and socio-demographic inequalities persist. At the same time, challenges exist to identify groups of women at high risk for non-participation, since it is likely that this is due to disadvantages on multiple social dimensions. This study, therefore, aimed to identify intersectional groups of women at higher risk of not attending BCS by comparing two analytical strategies: a) evidence-informed regression and b) decision tree-based regression. Participants were drawn from the German 2019 European Health Interview Survey (N = 23,001; 21.6% response rate). Two logistic regressions using cross-classification intersectional groups based on relevant PROGRESS-Plus characteristics adjusted by age were built. The evidence-informed approach selected relevant variables based on the literature and the decision tree approach on the best-performing tree. The first identified low-income women born outside Germany, living in rural areas and not cohabiting with their partner at higher risk of never attending BCS (OR = 9.48, p = 0.002), whereas the second, based on a Classification and Regression Tree (61.91% balanced accuracy), determined widowed women living alone, with children, with a partner and children, or in other arrangements, and residing in specific federal states (i.e. Bavaria, Brandenburg, Bremen, Hamburg, or Saarland) (OR = 3.43, p < 0.001). Compared to the evidence-informed regression, the decision tree-based regression yielded higher discriminatory accuracy (AUC = 0.6726 vs AUC = 0.6618) and added relevant nuances in the identification of at-risk intersectional groups, going beyond known inequality dimensions and, therefore, helping the inclusion of under-studied populations in breast cancer screening.

## Highlights

• In Germany, family status and regional inequalities in breast cancer screening exist.
• Decision trees aid variable selection and intersectional subgroup identification.
• Combining regression and decision tree approaches offers a comprehensive strategy.
• The authors suggest using diverse analytical strategies to answer a research question.

## Graphical abstract

**Figure:**
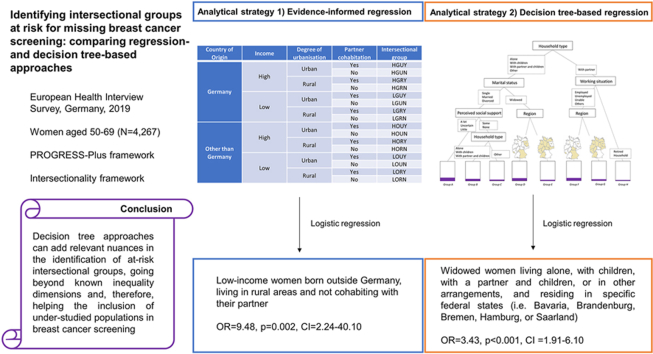

## 1 Introduction

Malignant neoplasm of the breast was the fifth leading cause of death among women in Germany in 2020, with 18,500 deaths, according to the last available statistics of the German Federal Statistical Office (Statistisches Bundesamt, 2024). Socioeconomic inequalities in mortality due to breast cancer (BC) have been documented in Germany both at the individual (Singer et al., 2017) and district level (Tetzlaff et al., 2023), where low-income women or women living in areas with higher levels of deprivation entail a higher mortality risk.

The European Commission encouraged Member States to implement organised screening programmes (OSP) in 2003, with invitations being sent out on a biannual basis to women aged between 50 and 69 (EC, 2022). Since then, numerous European studies have reported a decrease in breast cancer mortality rates (Poiseuil et al., 2023) and a reduction in inequalities in access to screening services (Palència et al., 2010).

Germany initiated the implementation of the OSP in 2005 and achieved full implementation by 2009 (Kooperationsgemeinschaft Mammographie, 2014). The participation rate following an invitation has fluctuated between 43% and 55% over the past two decades, failing to reach the 70% benchmark recommended by the European Commission (Cardoso et al., 2023; Kooperationsgemeinschaft Mammographie, 2023). Additionally, in 2020, 10.38% of the targeted women reported that they had never attended BCS in their lifetime (Eurostat, 2019).

Several studies have investigated the sociodemographic characteristics of women who are at higher risk of not participating in breast cancer screening (BCS) programmes. In the most recent international systematic review, Mottram et al. (2021) observed that migrant women, women with lower socioeconomic status, without home ownership, and those who experienced false positives had the lowest attendance rates (Mottram et al., 2021). In a scoping review of the German context, Pedrós Barnils et al. (2024) identified native women, women with lower incomes, women living in rural areas, and those not cohabiting with their partners as those with the lowest lifetime BCS attendance rates. However, the author also highlighted considerable heterogeneity in methods and, therefore, results (Pedrós Barnils et al., 2024).

Usually, inequalities in attendance are documented based on independent social dimensions. To explore correlations between social dimensions and BCS attendance, most studies incorporate variables deemed relevant (i.e. based on specific assumptions) into statistical models and then, in multivariate analyses, estimate the independent effect of each social dimension with the effects of other covariates held constant. However, as no individual can be defined by a single social dimension alone (Bowleg, 2012), it is unlikely that examining the independent effect of each social dimension will provide a comprehensive understanding of the inequalities in accessing cancer screenings.

Instead, individuals sit at the intersection of different social dimensions, and this needs to be considered when assessing who is at higher risk of not attending BCS. Methodologically and conceptually, the way the risk for not attending BCS of a person with a migration background and low educational attainment can be seen differently: either as the sum of (presumably) independent discrimination dimensions or as accounting for the discrimination of being a migrant from a lower social class simultaneously (Bowleg, 2008). It is, therefore, essential to employ a framework that allows to detect the inherent complexity of inequalities when attempting to understand the underlying factors influencing BCS attendance. The most appropriate approach is to adopt the framework of intersectionality (Crenshaw, 1989, pp. 57–80). Intersectionality theory, as first proposed by law scholar Kimberlé Crenshaw in 1989, posits that the experiences of discrimination (e.g. classism, racism) based on disadvantaged social positions (e.g. low social class, migration background) overlap and derive into unique experiences of discrimination (Crenshaw, 1991).

Over the past two decades, in the field of population health, quantitative intersectionality has given rise to new methodological approaches. The most commonly used methods for describing intersectional inequalities within a population range from simple cross-classification descriptions or regressions to methods that account for discriminatory accuracy (e.g. analysis of individual heterogeneity and discriminatory accuracy (AIHDA) and multilevel analysis of individual heterogeneity and discriminatory accuracy (MAIHDA)) or are data-driven (e.g. decision trees) (Bauer et al., 2021).

To build cross-classification regression and AIHDA or MAIHDA, the (potentially) relevant social dimensions are usually selected on the basis of the available evidence, and these dimensions are combined to identify intersectional subgroups. This is a deductive approach. In contrast, decision trees and analogous heuristic procedures employ an inductive methodology to identify which variables are most predictive of an outcome assuming non-linear relationships between the variables (Breiman et al., 2017). This enables a data-driven determination of the social dimensions that will constitute intersectional subgroups, often previously unnoticed (Mena et al., 2021). Decision trees have been applied as statistical exploratory tools for classification in population health (Eagle et al., 2022; Greene et al., 2019).

To the author's knowledge, no explicit comparisons between these approaches to identify intersectional inequalities in breast cancer screening have been conducted; besides, no study has employed an intersectional approach for reporting inequalities in breast cancer screening in Germany. Consequently, the present study aims to identify intersectional groups of women aged 50–69 who are at higher risk of never attending BCS in Germany comparing two analytical strategies: a) evidence-informed regression and b) decision tree-based regression.

## 2 Material and methods

### 2.1 European Health Interview Survey

For this analysis, we employed cross-sectional data from the European Health Interview Survey (EHIS) third wave conducted in Germany in 2019. The survey sample size was 23,001 respondents, corresponding to 21.6% of the invited participants (Jennifer Allen et al., 2021). EHIS is conducted every 5 years (6 since 2019) and focuses on individuals aged 15 and above residing in private households (European Parliament, December 16, 2008, 2018).

### 2.2 Primary outcome

The primary outcome of this study was self-reported breast cancer screening attendance via mammography at least once in a lifetime for women aged 50–69 in Germany. Responses were dichotomised, excluding those who indicated “unknown” or left the question unanswered to prevent uncertainty about whether the respondent reported never attending BCS (no = 0, yes = 1).

### 2.3 Explanatory variables

The explanatory variables to predict BCS derive from the PROGRESS-Plus characteristics: place of residence, race, ethnicity, culture and language, occupation, sex, education, socioeconomic status, social capital and plus (i.e. other potentially discriminatory factors) (Oliver et al., 2008). These variables have been widely used to disentangle social inequities in health (O'Neill et al., 2014; Coetzee et al., 2022).

*Place of Residence* was determined through the degree of urbanisation of the municipality and the specific region (*Bundesland*). The first variable was composed of three categories: cities (densely populated areas), towns and suburbs (intermediate-density areas), and villages (thinly populated areas). The second variable indicated the federal states (*Bundesländer*) in Germany.

*Race, ethnicity, culture, and language* were indicated by proxy variables: since the EHIS did not assess either of these explicitly. We selected the respondent's country of origin and nationality and then classified them as either born in Germany, in Europe or outside Europe. Although short at measuring complexities of identity, these variables have shown utility as ethnicity proxies in European countries where no information on race or ethnicity is gathered (Olczyk et al., 2014; Stronks et al., 2009).

*Occupation* was operationalised based on the respondents’ current working situation: in paid employment, unemployed, retired, unable to work, (unpaid) household work and others.

*Sex* (to identify as a female) was a prerequisite for participant inclusion in the analysis. *Gender* and *religion* were not captured by the EHIS.

*Education* was measured following the ISCED-2011 classification (UNESCO, 2012). Since only 6 participants had primary education or less, the first three categories were combined into “less than upper secondary education”.

*Socioeconomic status* was operationalised through household income and was divided into five quintile groups: the 20% with the lowest income were coded 1, and the 20% with the highest income were coded 5 (Dupré, 2020).

*Social capital* was considered through six variables: social network dimensions (none, 1–2, 3–5, 6 or more), perceived social support (a lot, some, uncertain, little, or no concern) and ease in available help (very easy, easy, possible, difficult, or very difficult). Further, three proxy variables were also included: marital status (single, married, legally separated/divorced or widowed), type of household (alone, with a partner, with a partner and children, with children, or other) indicating the availability of family support, and partner cohabitation (yes or no).

For the *Plus* dimension, the Global Activity Limitations Indicator (GALI), a self-report of the extent of limitation experienced in the last six months was considered, with possible answers: severely limited, mildly limited, or not limited (Robine & Jagger, 2003). Age (50–69 years old) was required to be included in the analysis and was treated as a confounder in the regression analyses.

### 2.4 Analytic approach

Descriptive analytics, including frequencies and percentages, were calculated for all variables. A complete case analysis was conducted, i.e. cases with missing data were excluded listwise. The total sample was restricted to women aged 50–69 (n = 5365). Among these women, those who did not respond on whether they underwent mammography (n = 15), their place of residence (n = 384), the degree of urbanisation of their place of residence (n = 213), the household's income (n = 122), their level of education (n = 14), their social network dimensions (n = 11), their perceived social support (n = 46), the available help (n = 81), the type of household (n = 56), their marital status (n = 13), their partnership cohabitation status (n = 30), their working situation (n = 10), their country of origin (n = 11), their citizenship (n = 7), their GALI (n = 7) were excluded. Hence, the final total sample size of the study was 4761 participants.

Sampling weights were not used in analyses, as the sampling weights provided in the German EHIS data were derived from variables included in the analyses (education, urbanisation and age), which could lead to multicollinearity and biased standard error estimation. We report sensitivity analyses applying the sampling weights in both analytical strategies in Appendix A and show the correlations between sampling weights and variables in the analyses in Appendix B. The central aim of this article was to compare the estimation of women at higher risk of never attending BCS using two different analytical strategies: (a) evidence-informed regression and (b) decision tree-based regression.

#### 2.4.1 Analytical strategy a: evidence-informed regression

The evidence-informed analytical strategy builds a full cross-classification matrix based on social dimensions identified as relevant in the literature. A recent scoping review pinpointed migration background, socioeconomic position (based on income), degree of urbanisation, and partner cohabitation as significant dimensions for BCS attendance prediction (Pedrós Barnils et al., 2024).

For this analysis, country of origin was dichotomised as born inside or outside Germany, income was dichotomised into low (categories 1 and 2) and high (categories 3, 4 and 5), degree of urbanisation was dichotomised in people living in cities (urban) and people living in towns, suburbs or rural areas (rural), and partner cohabitation was already a dichotomous variable (yes/no). The cross-classification of all social positions led to 16 intersectional groups: 2 (country of origin) ∗ 2 (income) ∗ 2 (degree of urbanisation) ∗ 2 (partner cohabitation) (Table 1).

**Table 1:** Evidence-informed intersectional groups on lifetime BCS attendance.

Country of origin	Income	Degree of urbanisation	Partner cohabitation	Intersectional group name
Germany	High	Urban	Yes	HGUY
			No	HGUN
		Rural	Yes	HGRY
			No	HGRN
	Low	Urban	Yes	LGUY
			No	LGUN
		Rural	Yes	LGRY
			No	LGRN
Other than Germany	High	Urban	Yes	HOUY
			No	HOUN
		Rural	Yes	HORY
			No	HORN
	Low	Urban	Yes	LOUY
			No	LOUN
		Rural	Yes	LORY
			No	LORN

For the purpose of comparison, univariate models were initially constructed for each of the four individual predictors and age. Next, a multivariable model that included all main effects was estimated.

Following this, a multivariate logistic regression with the full cross-classification matrix as the main predictor was performed to estimate the odds ratio (OR) of never attending BCS adjusted by age. Discriminatory accuracy (DA) was estimated through the area under the receiver operating characteristics curve (AUC) with a 95% confidence interval (CI), indicating how well each model discriminates between women attending and women never attending BCS. DA is considered absent or very small when 0.5 ≤AUC≤ 0.6, moderate when 0.6< AUC ≤0.7, large when 0.7< AUC ≤0.8 and very large AUC>0.8 (Axelsson Fisk et al., 2021). These statistical procedures were carried out using Stata version 17.0.

#### 2.4.2 Analytical strategy b: decision tree-based regressions

The second analytical strategy consisted of two steps. First, building an explorative decision tree with the total sample size to identify homogeneous subgroups of women at higher risk of never attending BCS in Germany. Second, performing a multivariate logistic regression using the outcome of the decision tree adjusted by age to estimate the OR of never attending BCS.

There is no consensus on which decision tree better operates on binary outcomes. In this study, we trained three different algorithms: Classification and Regression Tree (CART), Conditional Inference Tree (CIT) and C5.0. The CART algorithm makes splitting decisions based on the lowest gini impurity (or entropy) coefficient among all potential splits (i.e. every category or step of every variable) (Breiman et al., 2017). CART does not provide statistical significance measures and potentially overestimates the influence of variables with many categories. CIT addresses these limitations by utilising a formal statistical hypothesis in growing decision trees and mitigating variable selection bias by splitting the selection process into two steps (Hothorn et al., 2006). C5.0 uses the entropy coefficient of the imputed variables to generate splits plus adaptative boosting and winnowing (Max Kuhn, 2023).

All three decision tree algorithms (CART, CIT, C5.0) were built using the entire dataset (N = 4761) and the same subdivision of the data when performing cross-validation. Cost weights were applied to distribute the sums of weights equally for cases and non-cases, given the (relative) rareness of the outcome in the dataset (10.38% prevalence). Parameters were hypertuned and optimised by two performance measures: sensitivity (i.e. enhancing detection of positive cases) and the Area Under the Precision-Recall Curve (i.e. improving overall precision-recall performance for unbalanced datasets) (Saito & Rehmsmeier, 2015). Decision trees were grown using the *tune* function from the “mlr3tuning” optimisation R packages in R version 4.4.0. This package integrates essential packages for building CART “rpart” (Therneau et al., 2023), CIT “partykit” (Hothorn et al., 2006), and C5.0 “C50” (Max Kuhn, 2023).

After inductively identifying the best-performing decision tree, the final nodes were deductively used as predictors for a multivariate logistic regression adjusted by age, where the ORs and DA of the model were estimated. This statistical procedure was performed using the Stata version 17.0. Estimations, performance and interpretability of both analytical strategies were compared and discussed.

## 3 Results

### 3.1 Descriptive statistics of the sample

Summary descriptive statistics of the sample can be found in Table 2. The total sample size is 4761. Of those, 4267 attended BCS at least once in their lifetime, and 494 did not. Relative frequencies for never attending BCS among the different PROGRESS-Plus characteristics were assessed. As expected, women aged 65–69 had the lowest prevalence (6.55%), and women aged 50–54, had the highest prevalence (18.58%). For SES, women in the lowest quintile attended BCS the least (14.07%), and those in the highest quintile attended BCS the most (9.79%). Almost contradicting, women with the highest education attainment, doctoral or equivalent, attended BCS the least (14.75%) and women with bachelor or equivalent educational attainment the most (9.10%). Based on the country of origin, women born in Germany (10.56%) had the lowest attendance rates compared to women born elsewhere. However, women of another European nationality attended the least (12.05%) and women of German nationality the most (10.31%). Regarding the place of residence, women living in cities (11.16%), women living in Berlin (13.69%) and Saarland (13.39%) had the lowest BCS attendance rate.

**Table 2:** Descriptive PROGRESS-Plus characteristics on BCS attendance among targeted women in Germany. Relative frequencies per column and variable are displayed.

	Attended BCS (N = 4267)	Never attended BCS (N = 494)	Total (N = 4761)
Age			
50-54	916 (21.5%)	209 (42.3%)	1125 (23.6%)
55-59	1149 (26.9%)	116 (23.5%)	1265 (26.6%)
60-64	1104 (25.9%)	92 (18.6%)	1196 (25.1%)
65-69	1098 (25.7%)	77 (15.6%)	1175 (24.7%)
Income			
1Q	458 (10.7%)	75 (15.2%)	533 (11.2%)
2Q	687 (16.1%)	76 (15.4%)	763 (16.0%)
3Q	795 (18.6%)	89 (18.0%)	884 (18.6%)
4Q	1010 (23.7%)	111 (22.5%)	1121 (23.5%)
5Q	1317 (30.9%)	143 (28.9%)	1460 (30.7%)
Educational group			
Lower secondary or lower	219 (5.1%)	28 (5.7%)	247 (5.2%)
Upper secondary	1515 (35.5%)	170 (34.4%)	1685 (35.4%)
Post-secondary	609 (14.3%)	78 (15.8%)	687 (14.4%)
Bachelor or equivalent	1120 (26.2%)	112 (22.7%)	1232 (25.9%)
Master or higher^a^	804 (18.8%)	106 (21.4%)	900 (19.2%)
Country of origin			
Germany	3948 (92.5%)	466 (94.3%)	4414 (92.7%)
Outside of Germany^a^	319 (7.5%)	(28)^b^ (5.6%)	347 (7.3%)
Citizenship			
German or other^a^	4267 (100%)	494 (100%)	4761 (100%)
Degree of urbanisation			
City	1712 (40.1%)	215 (43.5%)	1927 (40.5%)
Town or suburb	1803 (42.3%)	210 (42.5%)	2013 (42.3%)
Rural area	752 (17.6%)	69 (14.0%)	821 (17.2%)
Region			
Baden-Württemberg	459 (10.8%)	(43)^b^ (8.7%)	502 (10.5%)
Bavaria	522 (12.2%)	71 (14.4%)	593 (12.5%)
Berlin/Brandenburg^a^	503 (11.8%)	83 (14.7%)	576 (12.1%)
Hesse	260 (6.1%)	(31)^b^ (6.3%)	291 (6.1%)
Lower Saxony/Bremen^a^	368 (8.8%)	(35)^b^ (7.1%)	403 (8.5%)
North Rhine-Westphalia/Rhineland-Palatinate^a^	991 (23.2%)	102 (20.6%)	1093 (22.9%)
Saarland	427 (10.0%)	66 (13.4%)	493 (10.4%)
Saxony/Saxony-Anhalt/Thuringia^a^	377 (8.8%)	(37)^b^ (7.4%)	414 (8.7%)
Schleswig-Holstein/Hamburg/Mecklenburg-Vorpommern^a^	360 (8.4%)	(36)^b^ (7.2%)	397 (8.3%)
Quality of social network			
1–2 or less^a^	557 (13.1%)	82 (16.6%)	639 (13.5%)
3-5	2086 (48.9%)	252 (51.0%)	2338 (49.1%)
>6	1624 (38.1%)	160 (32.4%)	1784 (37.5%)
Perceived social support			
A lot	930 (21.8%)	116 (23.5%)	1046 (22.0%)
Some	2529 (59.3%)	260 (52.6%)	2789 (58.6%)
Uncertain	523 (12.3%)	77 (15.6%)	600 (12.6%)
Little or none^a^	275 (6.7%)	(41)^b^ (8.3%)	326 (6.8%)
Available help			
Very easy	1414 (33.1%)	165 (33.4%)	1579 (33.2%)
Easy	1702 (39.9%)	176 (35.6%)	1878 (39.4%)
Possible	726 (17.0%)	93 (18.8%)	819 (17.2%)
Difficult	291 (6.8%)	(34)^b^ (6.9%)	325 (6.8%)
Very difficult	134 (3.1%)	(26)^b^ (5.3%)	160 (3.4%)
Marital status			
Single	481 (11.3%)	95 (19.2%)	576 (12.1%)
Married	2706 (63.4%)	267 (54.0%)	2973 (62.4%)
Widowed	449 (10.5%)	(37)^b^ (7.5%)	486 (10.2%)
Divorced	631 (14.8%)	95 (19.2%)	726 (15.2%)
Type of household			
Alone	1219 (28.6%)	168 (34.0%)	1387 (29.1%)
With children	145 (3.4%)	(21) (4.3%)	166 (3.5%)
With a partner	2100 (49.2%)	172 (34.8%)	2272 (47.7%)
With a partner and children	435 (10.2%)	86 (17.4%)	521 (10.9%)
Other	368 (8.6%)	(47)^b^ (9.5%)	415 (8.7%)
Working situation			
In paid employment	2531 (59.3%)	332 (67.2%)	2863 (60.1%)
Unemployed/Others^a^	135 (3.1%)	(20)^b^ (4.0%)	155 (3.3%)
Retired	1258 (29.5%)	90 (18.2%)	1348 (28.3%)
Household work (unpaid)	181 (4.2%)	(25)^b^ (5.1%)	206 (4.3%)
Unable	162 (3.8%)	(27)^b^ (5.5%)	189 (4.0%)
Partner cohabitation			
Yes	2767 (64.8%)	280 (56.7%)	3047 (64.0%)
No	1500 (35.2%)	214 (43.3%)	1714 (36.0%)
Experienced limitation			
Severely limited	320 (7.5%)	(44) (8.9%)	364 (7.6%)
Mildly limited	1339 (31.4%)	137 (27.7%)	1476 (31.0%)
Not limited	2608 (61.1%)	313 (63.4%)	2921 (61.4%)

a Multiple categories were displayed collapsed when cell sizes <20 observations to avoid re-identifiability according to EHIS anonymisation rules.

b Cells containing between 20 and 49 observations are individually flagged according to EHIS anonymisation rules.

When considering social capital, the highest prevalence of never attending BCS was among those with no social network (13.35%), those with little perceived social support (13.36%), and those who find it very difficult to get help from neighbours (16.25%).

Single women (16.49%) showed the highest rates of never attending BCS among all marital statuses. Women living with a partner and children (16.51%), women unable to work (13.76%), unemployed women (13.68%), and women not cohabiting with a partner (12.49%) displayed the highest prevalences. Lastly, severely limited women (12.09%) had the lowest attendance rates among their PROGRESS-Plus dimension.

### 3.2 Intersectional inequities in BCS attendance in Germany

#### 3.2.1 Analytical strategy a: evidence-informed regression

Based on a recent scoping review (Pedrós Barnils et al., 2024), four PROGRESS-Plus variables were relevant for predicting lifetime BCS attendance: migration background, income, urbanisation degree and partnership cohabitation (Pedrós Barnils et al., 2024).

Univariate logistic regression analyses separately estimated the effects of these four variables (Table 3). Only cohabitation significantly predicted BCS attendance, with women living alone having higher odds of never attending. Age also had a significant relationship with BCS attendance.

**Table 3:** Univariate logistic regression on never attending BCS in Germany.

Sociodemographic variables	OR	95% CI	R^2^ model	AUC model
Income				
High	1			
Low	1.20	(0.98–1.47)	0.0010	0.5187
Country of origin				
Germany	1			
Not Germany	0.74	(0.50–1.11)	0.0007	0.5090
Degree of urbanisation				
Urban	1			
Rural	0.87	(0.72–1.05)	0.0007	0.5170
Partner cohabitation				
Yes	1			
No	1.41∗∗∗	(1.17–1.70)	0.0039	0.5408
Age				
50–54	1			
55–59	0.44∗∗∗	(0.35–0.56)		
60–64	0.37∗∗∗	(0.28–0.47)		
65–69	0.31∗∗∗	(0.23–0.40)	0.0317	0.6225

Multivariate logistic regression was performed to capture the effects of each predictor when adjusting for covariates and age (Table 4). Here, the only relationship that showed a statistically significant relationship with BCS attendance was partner cohabitation, with 1.45 higher odds (p < 0.001) for women not cohabitating with their partners.

**Table 4:** Multivariate logistic regression on never attending BCS in Germany (main effects model).

Sociodemographic variables	OR	95% CI
Income		
High	1	
Low	1.21	(0.98–1.49)
Country of origin		
Germany	1	
Not Germany	0.68	(0.46–1.02)
Degree of urbanisation		
Urban	1	
Rural	0.91	(0.75–1.11)
Partner cohabitation		
Yes	1	
No	1.45∗∗∗∗	(1.19–1.76)
Age		
50–54	1	
55–59	0.43 ∗∗∗	(0.34–0.56)
60–64	0.36 ∗∗∗	(0.28–0.47)
65–69	0.29 ∗∗∗	(0.22–0.38)
R^2^	0.0394	
AUC-ROC	0.6539	

A complete case analysis only based on the variables would have resulted in 300 more participants, but the results do not change meaningfully – see Appendix C.

**Fig. 1:**
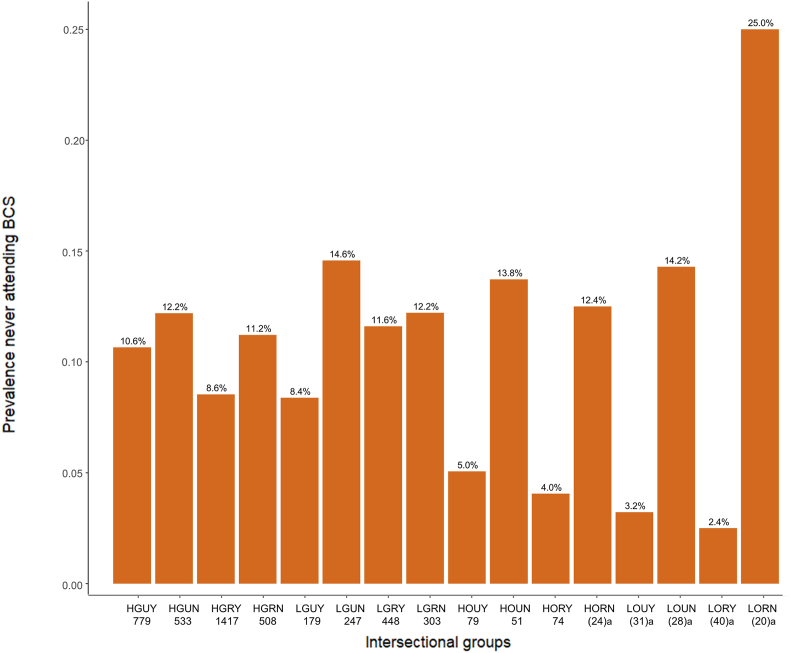
Prevalence and size across the sixteen evidence-informed intersectional groups. ^a^ Cells containing between 20 and 49 observations are individually flagged according to EHIS anonymisation rules.

Sixteen intersectional groups were created based on the combination of the four variables identified in the literature. Fig. 1 depicts the size and prevalence of each group.

**Fig. 2:**
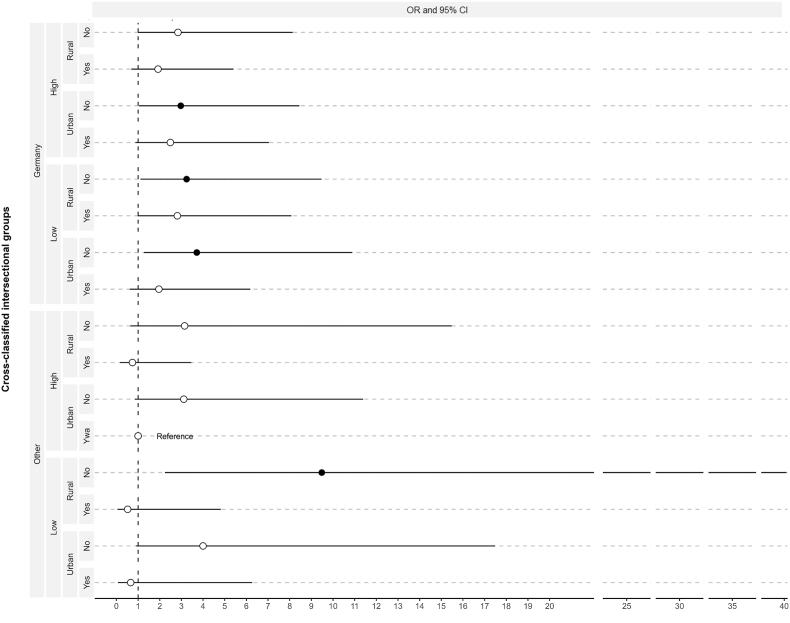
Odds Ratio (OR) with evidence-informed intersectional groups on never attending BCS in Germany.

Following this, an unweighted logistic regression was performed (Table 5, Fig. 2). As a reference group, we chose the one expected to have the highest attendance rate - based on the multivariate regression and Pedrós Barnils et al. (2024) (Pedrós Barnils et al., 2024) - high-income women born outside Germany, living in urban areas with a partner (HOUY).

**Table 5:** Full cross-classified multivariate logistic regression with evidence-informed intersectional groups.

	OR	95% CI
Intersectional groups		
HOUY	1	
HGUY	2.49	(0.88–7.04)
HGUN	2.97∗	(1.04–8.45)
HGRY	1.92	(0.69–5.40)
HGRN	2.84	(0.99–8.14)
LGUY	1.96	(0.62–6.18)
LGUN	3.71∗	(1.27–10.89)
LGRY	2.81	(0.98–8.08)
LGRN	3.24∗	(1.11–9.47)
HOUN	3.11	(0.85–11.39)
HORY	0.74	(0.16–3.46)
HORN	3.15	(0.64–15.48)
LOUY	0.66	(0.07–6.26)
LOUN	4.00	(0.91–17.49)
LORY	0.52	(0.05–4.82)
LORN	9.48∗∗	(2.24–40.10)
Age		
50–54	1	
55–59	0.43∗∗∗	(0.33–0.54)
60–64	0.35∗∗∗	(0.27–0.45)
65–69	0.29∗∗∗	(0.22–0.38)
R^2^	0.0445	
AUC-ROC	0.6618	

∗p-value <0.05; ∗∗p-value <0.01; ∗∗∗ p-value <0.001.

HGUY - high-income, born in Germany, urban, cohabitation.

HGUN - high-income, born in Germany, urban, no cohabitation.

HGRY - high-income, born in Germany, rural, cohabitation.

HGRN - high-income, born in Germany, rural, no cohabitation.

LGUY - low-income, born in Germany, urban, with cohabitation.

LGUN - low-income, born in Germany, urban, no cohabitation.

LGRY - low-income, born in Germany, rural, cohabitation.

LGRN - low-income, born in Germany, rural, no cohabitation.

HOUY - high-income, born outside Germany, urban, cohabitation.

HOUN - high-income, born outside Germany, urban, no cohabitation.

HORY - high-income, born outside Germany, rural, cohabitation.

HORN - high-income, born outside Germany, rural, no cohabitation.

LOUY - low-income, born outside Germany, urban, cohabitation.

LOUN - low-income, born outside Germany, urban, no cohabitation.

LORY - low-income, born outside Germany, rural, cohabitation.

LORN - low-income, born outside Germany, rural, no cohabitation.

Four intersectional groups were significantly associated with never attending BCS. Low income women not born in Germany and living in rural areas with no partner (LORN) showed the highest odds (OR = 9.48, p = 0.002). The confidence intervals for all these estimations were rather wide, increasing the uncertainty of the predicted estimations. The DA of the full cross-classification model was moderated (AUC = 0.6618) and 0.0079 points higher than the main effects model. That indicates that the regression with intersectional groups discriminates slightly better between women attending or never attending BCS than the main effects model.

#### 3.2.2 Analytical strategy b: decision tree-based regression

**Fig. 3:**
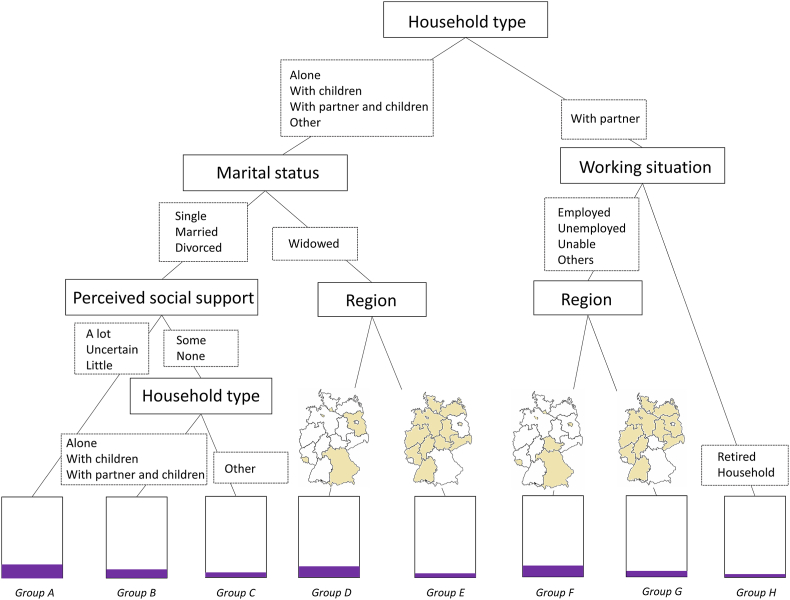
CART decision tree on never attending BCS in Germany.

Out of the three algorithms, CART (cp= 0.006713025 and maxdepth = 4) showed the highest sensitivity and balanced accuracy performance. For more information on the hypertuned models, see Appendix D. The inner performance (i.e. evaluated on trained data) of CART was: 72.47% sensitivity, 51.35% specificity, 61.91% balanced accuracy, 14.71% positive predictive value and 94.15% negative predictive value. The moderate sensitivity suggests reasonable confidence in CART detecting women not attending BCS. However, the low specificity suggests small confidence in CART to identify negative cases (i.e. women attending BCS). The small positive predictive value indicates that many cases classified as positive (i.e. not attending BCS) are false positives. Nevertheless, the high negative predictive value indicates very few false negatives and, therefore, very high confidence that those cases classified as negative are negative (i.e. not assuming that a woman is attending BCS when she is not). Fig. 3 and Table 6 show the final decision tree and the emerged intersectional groups.

**Table 6:** Intersectional groups on never attending BCS in Germany based on CART.

Group	Intersectional groups	Rank^a^	Size, Prevalence
H	Women living with a partner, retired or doing unpaid household work	1	N = 882 Pr = 0.0454
E	Widowed women living alone, with children, with a partner and children or other arrangements, residing in Baden-Württemberg, Berlin, Hesse, Mecklenburg-Vorpommern, Lower Saxony, North Rhine-Westphalia, Rhineland-Palatinate, Saxony, Saxony-Anhalt, and Schleswig-Holstein or Thuringia	2	N = 316 Pr = 0.0506
C	Single, married or divorced women living in other living arrangements, with some or no perceived social support	3	N = 211 Pr = 0.0616
G	Women living with a partner, who are either employed, unemployed, unable to work, or in other categories, and residing in Baden-Württemberg, Brandenburg, Hesse, Mecklenburg-Vorpommern, Lower Saxony, North Rhine-Westphalia, Rhineland-Palatinate, Saxony, Saxony-Anhalt, or Schleswig-Holstein	4	N = 918 Pr = 0.0730
B	Single, married or divorced women living alone, with children, with a partner and children, with some or no perceived social support	5	N = 953 Pr = 0.1301
F	Women living with a partner who are either employed, unemployed, unable to work, or in other working categories and residing in Bavaria, Berlin, Bremen, Hamburg, Saarland or Thuringia	6	N = 472 Pr = 0.1377
D	Widowed women living alone, with children, with a partner and children or other arrangements, residing in Bavaria, Brandenburg, Bremen, Hamburg, or Saarland	7	N = 136 Pr = 0.1471
A	Single, married or divorced women living alone, with children, with a partner and children or other arrangements, with little, uncertain or a lot of perceived social support	8	N = 873 Pr = 0.1707

CART identified household type, marital status, working situation, region and perceived social support as relevant variables. The first splitting point, the root node, is the household type, where women living with a partner are split from all other household types. Women living with a partner are further split into working situations. Here, women retired or doing unpaid household work form a final node (Group H; N = 882; Pr = 0.0454), and women employed, unemployed, unable to work, or others further split based on their region (Group G; N = 918; Pr = 0.0730; Group F; N = 472; Pr = 0.1377).

Women living alone, with children, with a partner and children or in other arrangements are further split based on marital status. Here, widowed women are separated from single, married or divorced women. Widowed women were lastly split based on their region (Group E; N = 316; Pr = 0.0506; Group D; N = 136; Pr = 0.1471). On the other hand, single, married or divorced women further split based on their perceived social support. Those with some or no perceived social support are separated from those with little, uncertain or a lot of perceived social support, who form a final node (Group A; N = 873; Pr = 0.1707). The first group split one last time based again on their type of household: living alone, with children, or with a partner and children (Group B; N = 953; Pr = 0.1301), and other arrangements (Group C; N = 211; Pr = 0.0616).

**Fig. 4:**
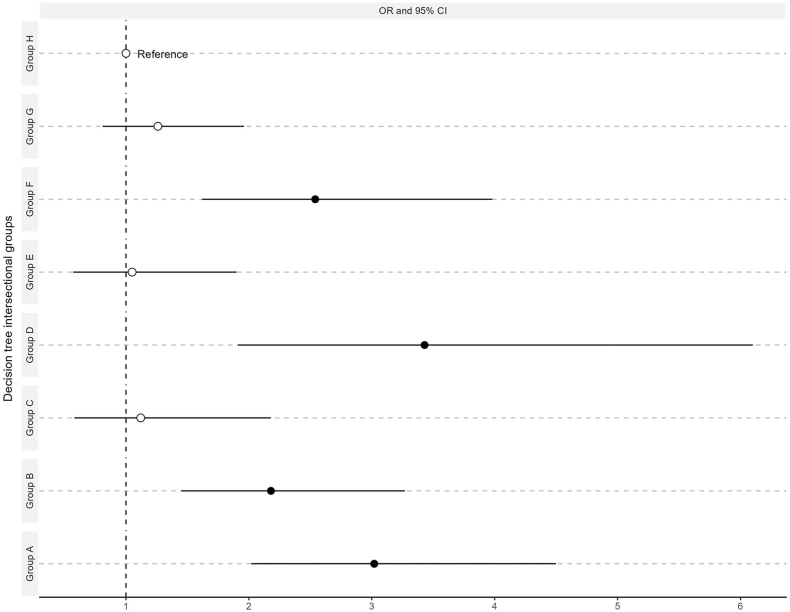
Odds Ratios (OR) from CART intersectional groups on never attending BCS in Germany.

An unweighted logistic regression with CART intersectional groups adjusted by age was carried out using the group with the lowest never-attended BCS prevalence (Group H) as the reference category (Table 7, Fig. 4).

**Table 7:** Multivariate logistic regression with CART intersectional groups on never attending BCS in Germany.

	OR	95% CI
CART intersectional groups		
A	3.02∗∗∗	(2.02–4.50)
B	2.18∗∗∗	(1.45–3.27)
C	1.12	(0.58–2.18)
D	3.43∗∗∗	(1.91–6.10)
E	1.05	(0.57–1.90)
F	2.54∗∗∗	(1.62–3.98)
G	1.26	(0.81–1.96)
H	1	
Age		
50–54	1	
55–59	0.49∗∗∗	(0.37–0.62)
60–64	0.46∗∗∗	(0.35–0.60)
65–69	0.44∗∗∗	(0.32–0.60)
R^2^	0.0534	
AUC-ROC	0.6726

After adjusting by age, four CART intersectional groups showed a statistically significant difference compared to group H. Group D showed the highest odds of never attending BCS (OR = 3.43; p < 0.001), and Group E the lowest odds (OR = 1.05; p = 0.88).

The total DA of the model CART was moderate (AUC = 0.6726). This value is 0.0108 points higher than the evidence-informed regression, indicating better discriminatory accuracy than the evidence-informed approach.

## 4 Discussion

### 4.1 Summary of findings

This study aimed to identify intersectional groups of women aged 50–69 at higher risk of never attending BCS in Germany comparing two different analytical strategies: evidence-informed regression and decision tree-based regression.

The evidence-informed approach identified low-income women who were not born in Germany, residing in rural areas and are not cohabitating with their partner as those at the highest risk of never attending BCS. In contrast, the decision tree-based approach yielded additional insights regarding specific regions of residence and family status. In this regard, the highest-risk group comprised women living alone, with children, with a partner and children, or in other arrangements, residing in Bavaria, Brandenburg, Bremen, Hamburg, or Saarland.

The evidence-informed intersectional group matrix presented low-income women not born in Germany living in rural areas and cohabiting with a partner as those with the lowest prevalence of never attending BCS and low-income women not born in Germany living in rural areas and not cohabiting with a partner as those with the highest prevalence. These two groups differ solely on partnership cohabitation, illustrating a classical intersectional hypothesis: the contingency of inequities, whereby discrimination experienced in a specific social position depends on its interactions with other social positions. Cohabitation with a partner acts as a determining factor for low-income women not born in Germany and living in rural areas on their likelihood of attending BCS.

The decision tree-based approach also identified household type as a relevant variable, revealing that women living with a partner generally had a lower risk of never attending BCS than those in other living arrangements. Several authors have previously conveyed the importance of partnership cohabitation and breast cancer screening attendance (Hanske et al., 2016; Missinne et al., 2013). Furthermore, living arrangements seem to play a role for those with some or no social support. The risk of never attending BCS was found to be half that of women with some or no social support who were living in other arrangements, compared to those living alone, with children, or with a partner and children. Furthermore, the role of perceived social support in the intersectional group identified as relevant (i.e. single, married or divorced women living alone, with children, with a partner and children or in other arrangements) is unclear, as previously noted in the literature (Allen et al., 2008; Manjer et al., 2015).

Lastly, the decision tree split by federal states twice in its third node. In both splits, women residing in Bavaria, Bremen, Hamburg, and Saarland indicate a higher risk of never attending BCS. These four federal states have been identified in other studies as having lower BCS attendance after invitation (Groβmann et al., 2023), reinforcing the higher compliance with preventive behaviours in former East Germany compared to the West. The use of the decision tree facilitated the identification of regional disparities among specific intersectional subgroups of women that would otherwise have remained unnoticed.

### 4.2 Comparison of regression- and decision tree-based approaches

The interpretability of the decision tree-based regression was slightly enhanced compared to the cross-classified regressions since it entailed fewer intersectional groups. Moreover, this reduction in dimensions did not entail a loss of information. On the contrary, the discriminatory accuracy of the model was slightly higher than the evidence-informed regression. Furthermore, the confidence intervals of the decision tree-based regression estimations are reduced (i.e. smaller variance), suggesting a more precise estimation of effect sizes.

Nevertheless, in this study no clearly discernible pattern of inequalities emerged among the PROGRESS-Plus characteristics, strengthening the heterogeneous findings reported by Pedrós Barnils et al. (2024) (Pedrós Barnils et al., 2024). Additional variables beyond the categorisation of sociodemographic factors, such as process-oriented variables (e.g. unpaid household work), could be explored to assess their relationship with BCS attendance.

This article does not aim to defend the use of any approach over another. As the “no-free-lunch theorem” in the machine learning literature often states, no single model works best in all scenarios (Ho & Pepyne, 2002). Nonetheless, this article encourages peer colleagues to evaluate different analytical strategies to answer their research question, while being aware of the advantages and disadvantages offered by each approach.

The evidence-informed approach synthesises existing research to identify variables that are relevant to BCS attendance, making a normative decision on which axis of inequality to explore. However, using repeatedly explored social dimensions may result in the stigmatisation of certain collectives and the under-exploration of others (North & Fiske, 2014; Turan et al., 2019). Moreover, recommendations can only be formulated based on analysed social dimensions. Consequently, a potentially biased selection of variables can result in biased recommendations for developing interventions.

Conversely, the decision tree-based approach uses statistical algorithms to inductively identify patterns and relationships from the dataset (Venkatasubramaniam et al., 2017). This approach is advantageous in revealing combinations of social dimensions not previously identified or explored, enabling more targeted interventions. Nevertheless, decision trees are susceptible to data quality, and their hierarchical structure might produce spurious results (i.e. the initial split has a significant impact on subsequent splits) (Apté & Weiss, 1997). Moreover, given the uncommon application of decision trees in public health, no standardised procedures are yet defined, hence, many decisions are left to the discretion of the researcher (i.e. researcher bias).

From a quantitative intersectionality perspective, the decision tree approach yields certain advantages for answering the question*, “Who is at higher risk of never attending BCS?“*. The regression with a full cross-classification based on evidence-identified variables inevitably results in a loss of information due to the category simplification required to build the matrix (McCall, 2005; Pedros Barnils et al., 2020). To maintain cells with sufficient size to preserve statistical power, variable categories are dichotomised, compromising the possibility of identifying non-linear patterns amid these categories (Pedrós Barnils & Schüz, 2024). Decision trees allow for high dimensionality in the included variables (i.e. without risk of multicollinearity) and their categories (i.e. no need for dichotomisation).

### 4.3 Strengths and limitations

To the authors’ knowledge, this is the first study to compare regression- and decision tree-based approaches for identifying intersectional subgroups of women at higher risk of not attending BCS. However, the study is not without limitations, in particular the cross-sectional design of the survey, which impedes any causal inference from being drawn, and its self-report methodology, which may introduce response bias. The response rate for EHIS wave 3 in Germany was 21.6%, highlighting the necessity for caution when interpreting findings from studies utilising this dataset. Lastly, previous studies have indicated that EHIS may underestimate disparities in access to screening programmes (Molina-Barceló et al., 2021).

## 5 Conclusion

The combination of regression and decision tree-based approaches provides a comprehensive strategy for identifying intersectional groups at higher risk of an outcome. In this study, the evidence-informed regression identified that low-income women who were not born in Germany lived in rural areas, and did not cohabit with their partner as being at the highest risk of never attending BCS. Conversely, the decision tree-based approach identified widowed women living alone, with children, with a partner and children, or in other arrangements, and residing in specific federal states (i.e. Bavaria, Brandenburg, Bremen, Hamburg, or Saarland) as the highest risk group. The decision tree-based approach slightly outperformed the regression-based approach in its overall performance and interpretability and added a nuanced, data-driven layer of analysis, that enhances the overall understanding of the PROGRESS-Plus characteristics that determine BCS attendance in Germany.

## CRediT authorship contribution statement

**Núria Pedrós Barnils:** Writing – original draft, Visualization, Methodology, Investigation, Formal analysis, Data curation, Conceptualization. **Benjamin Schüz:** Writing – review & editing, Validation, Supervision, Methodology, Conceptualization.

## Ethical statement

This study does not require ethical approval as it is a secondary analysis of de-identified data. Access to the data was granted by Eurostat, the European body for Statistics, and all authors have signed the individual confidentiality declaration following Regulation (EC) No 223/2009 (European Parliament, 2009).

## Financial disclosure statement

This research did not receive any specific grant from funding agencies in the public, commercial, or not-for-profit sectors. Open-access funding is provided by Bremen University.

## Declaration of interest statement

The results and conclusions are mine and not those of Eurostat, the European Commission or any of the national statistical authorities whose data have been used.

## Data Availability

The authors do not have permission to share data.

## Abbreviations

AUC: Area Under the receiver operating characteristics Curve
BCS: Breast cancer screening
CART: Classification And Regression Tree
CI: Confidence Interval
CIT: Conditional Inference Tree
DA: Discriminatory accuracy
EHIS: European Health Interview Survey
HGRN: High-income, born in Germany, rural, no cohabitation
HGRY: High-income, born in Germany, rural, cohabitation
HGUN: High-income, born in Germany, urban, no cohabitation
HGUY: High-income, born in Germany, urban, cohabitation
HORN: High-income, born outside Germany, rural, no cohabitation
HORY: High-income, born outside Germany, rural, cohabitation
HOUN: High-income, born outside Germany, urban, no cohabitation
HOUY: High-income, born outside Germany, urban, cohabitation
LGRN: Low-income, born in Germany, rural, no cohabitation
LGRY: Low-income, born in Germany, rural, cohabitation
LGUN: Low-income, born in Germany, urban, no cohabitation
LGUY: Low-income, born in Germany, urban, with cohabitation
LORN: Low-income, born outside Germany, rural, no cohabitation
LORY: Low-income, born outside Germany, rural, cohabitation
LOUN: Low-income, born outside Germany, urban, no cohabitation
LOUY: Low-income, born outside Germany, urban, cohabitation
OR: Odds Ratio
p: p-value
