# Supplementary material for: Identifying intersectional groups at risk for missing breast cancer screening: Comparing regression- and decision tree-based approaches
Source: SSM Popul Health. 2024 Dec 9;29:101736. doi: 10.1016/j.ssmph.2024.101736 (PMC11699213; doi:10.1016/j.ssmph.2024.101736)
Supplement: Multimedia component 1 [file mmc1.docx]

**Appendix A. Sensitivity analysis weighted logistic regressions Approach a and Approach b**

**Approach a**

|  | Odds ratio | Robust  std. err. | z | P>\|z\| | 95% CI | |
| --- | --- | --- | --- | --- | --- | --- |
| Int. groups |  |  |  |  |  |  |
| HOUY | 3.667521 | 2.469414 | 1.93 | 0.054 | .980036 | 13.72471 |
| HGUY | 6.127641 | 4.203189 | 2.64 | 0.008 | 1.597426 | 23.5053 |
| HGUN | 3.064387 | 2.054391 | 1.67 | 0.095 | .8235512 | 11.40241 |
| HGRY | 2.854078 | 1.976236 | 1.51 | 0.130 | .7346364 | 11.08815 |
| HGRN | 3.279509 | 2.359808 | 1.65 | 0.099 | .8004185 | 13.43694 |
| LGUY | 4.482464 | 3.094689 | 2.17 | 0.030 | 1.158372 | 17.34545 |
| LGUN | 4.332695 | 2.910457 | 2.18 | 0.029 | 1.161368 | 16.16392 |
| LGRY | 4.462574 | 3.058048 | 2.18 | 0.029 | 1.164894 | 17.0956 |
| LGRN | 3.593489 | 3.56475 | 1.29 | 0.197 | .5141885 | 25.11368 |
| HOUN | 1.462867 | 1.315203 | 0.42 | 0.672 | .2511452 | 8.520883 |
| HORY | 1.517906 | 1.914314 | 0.33 | 0.741 | .1281587 | 17.978 |
| HORN | .6890104 | .6977404 | -0.37 | 0.713 | .0946759 | 5.014323 |
| LOUY | 5.500932 | 4.617175 | 2.03 | 0.042 | 1.061659 | 28.50279 |
| LOUN | 1.456327 | 1.812355 | 0.30 | 0.763 | .127047 | 16.69373 |
| LORY | 34.40762 | 31.35341 | 3.88 | 0.000 | 5.767803 | 205.2574 |
|  |  |  |  |  |  |  |
| Age |  |  |  |  |  |  |
| 55-59 | .4687808 | .0797029 | -4.46 | 0.000 | .3359287 | .6541729 |
| 60-64 | .4186158 | .0838502 | -4.35 | 0.000 | .2826944 | .6198894 |
| 65-69 | .2841499 | .0622501 | -5.74 | 0.000 | .184957 | .4365402 |
|  |  |  |  |  |  |  |
| _cons | .0543577 | .0355643 | -4.45 | 0.000 | .0150782 | .1959619 |

**Approach b**

|  | Odds ratio | Robust  std. err. | z | P>\|z\| | 95% CI | |
| --- | --- | --- | --- | --- | --- | --- |
| CART  Int. groups |  |  |  |  |  |  |
| A | 2.227276 | .7163157 | 2.49 | 0.013 | 1.185819 | 4.183401 |
| B | 1.565419 | .496195 | 1.41 | 0.157 | .8410526 | 2.913656 |
| C | 1.370717 | .7028176 | 0.62 | 0.539 | .5017705 | 3.744471 |
| D | 3.178308 | 1.395339 | 2.63 | 0.008 | 1.344318 | 7.514328 |
| E | .7809968 | .3813354 | -0.51 | 0.613 | .2999405 | 2.03359 |
| F | 2.008606 | .6987073 | 2.00 | 0.045 | 1.015778 | 3.971833 |
| G | 1.068804 | .3505545 | 0.20 | 0.839 | .5619716 | 2.032739 |
|  |  |  |  |  |  |  |
| Age |  |  |  |  |  |  |
| 55-59 | .5414562 | .0981237 | -3.39 | 0.001 | .3795832 | .7723598 |
| 60-64 | .5404843 | .1161503 | -2.86 | 0.004 | .3546986 | .8235815 |
| 65-69 | .391003 | .1034453 | -3.55 | 0.000 | .2327999 | .6567156 |
|  |  |  |  |  |  |  |
| _cons | .1225309 | .0400627 | -6.42 | 0.000 | .064556 | .2325704 |
