# Supplementary material for: Identifying intersectional groups at risk for missing breast cancer screening: Comparing regression- and decision tree-based approaches
Source: SSM Popul Health. 2024 Dec 9;29:101736. doi: 10.1016/j.ssmph.2024.101736 (PMC11699213; doi:10.1016/j.ssmph.2024.101736)
Supplement: Multimedia component 2 [file mmc2.docx]

**Appendix B. Pairwise correlation table of all variables in Approaches a and b with weights**

|  | Sampling weights | p-value |
| --- | --- | --- |
| Age | -0.1280 | 0.0000 |
| Partner cohabitation | 0.1447 | 0.0000 |
| Degree of urbanisation | 0.1380 | 0.0000 |
| Country of origin | 0.0053 | 0.7163 |
| Income | -0.2649 | 0.0000 |
| Perceived social support | 0.0959 | 0.0000 |
| Type of household | -0.0986 | 0.0000 |
| Working situation | 0.0321 | 0.0270 |
| Region | -0.0502 | 0.0005 |
| Marital status | 0.0647 | 0.0000 |
