# Supplementary material for: Identifying intersectional groups at risk for missing breast cancer screening: Comparing regression- and decision tree-based approaches
Source: SSM Popul Health. 2024 Dec 9;29:101736. doi: 10.1016/j.ssmph.2024.101736 (PMC11699213; doi:10.1016/j.ssmph.2024.101736)
Supplement: Multimedia component 3 [file mmc3.docx]

**Appendix C. Approach a based on complete case analysis only from included variables**

**Table A.** Univariate logistic regression on never attending BCS in Germany

| Sociodemographic variables | OR | 95% CI | R2 model | AUC model |
| --- | --- | --- | --- | --- |
| Socioeconomic position (income)  High  Low | 1  1.12 | (0.94-1.34) | 0.0005 | 0.5145 |
| Migration background  Germany  Not Germany | 1  0.73 | (0.51-1.05) | 0.0009 | 0.5102 |
| Degree of urbanisation  Urban  Rural | 1  0.88 | (0.73-1.06) | 0.0005 | 0.5154 |
| Partnership cohabitation  Yes  No | 1  1.40*** | (1.17-1.67) | 0.0038 | 0.5402 |
| Age  50-54  55-59  60-64  65-69 | 1  0.42***  0.38***  0.32*** | (0.33-0.53)  (0.30-0.49)  (0.25-0.41) | 0.0310 | 0.6179 |

**Table B.** Multivariate logistic regressionon never attending BCS in Germany (main effects model)

| Sociodemographic variables | OR | 95% CI |
| --- | --- | --- |
| Socioeconomic position (income)  High  Low | 1  1.11 | (0.92-1.33) |
| Migration background  Germany  Not Germany | 1  0.72 | (0.49-1.05) |
| Urbanisation degree  Urban  Rural | 1  0.93 | (0.77-1.12) |
| Cohabitation  Yes  No | 1  1.46*** | (1.21-1.77) |
| Age  50-54  55-59  60-64  65-69 | 1  0.42 ***  0.36 ***  0.29 *** | (0.33-0.53)  (0.28-0.46)  (0.22-0.38) |
|  |  |  |
| R^2^ | 0.0394 |  |
| AUC-ROC | 0.6512 |  |

**Table C.** Full cross-classified multivariate logistic regression with literature-based intersectional groups on never attending BCS in Germany

| Intersectional groups | OR | 95% CI |
| --- | --- | --- |
| HOUY | 1 |  |
| HGUY | 2.78 | (0.88-9.18) |
| HGUN | 3.35* | (1.00-11.20) |
| HGRY | 1.96 | (0.60-6.41) |
| HGRN | 3.17 | (0.94-10.67) |
| LGUY | 2.16 | (0.63-7.38) |
| LGUN | 3.36* | (1.00-11.17) |
| LGRY | 2.71 | (0.82-8.89) |
| LGRN | 3.24 | (0.98-10.76) |
| HOUN | 2.12 | (0.44-10.18) |
| HORY | 1.06 | (0.20-5.52) |
| HORN | 2.25 | (0.34-14.74) |
| LOUY | 0.72 | (0.11-4.51) |
| LOUN | 5.60** | (1.40-22.29) |
| LORY | 0.63 | (0.10-3.97) |
| LORN | 9.11** | (2.19-37.85) |
| Age  50-54  55-59  60-64  65-69 | 1  0.42***  0.35***  0.28*** | (0.33-0.53)  (0.27-0.45)  (0.22-0.38) |
|  |  |  |
| R^2^ | 0.0462 |  |
| AUC-ROC | 0.6592 |  |
