# Supplementary material for: Identifying intersectional groups at risk for missing breast cancer screening: Comparing regression- and decision tree-based approaches
Source: SSM Popul Health. 2024 Dec 9;29:101736. doi: 10.1016/j.ssmph.2024.101736 (PMC11699213; doi:10.1016/j.ssmph.2024.101736)
Supplement: Multimedia component 4 [file mmc4.docx]

**Appendix D. Evaluation of hyper-tuned decision trees (Approach b)**

When choosing which decision tree is better for classifying women who will and women who will not attend breast cancer screening (BCS), their performance is considered. Here, a performance measure, or several, must be chosen and justified.

Decision trees are built in the mlr3 ecosystem, which allows the decision tree to grow based on one (auto_tuner function) or two (tune function) performance measures.

For our research question, since we have defined women who did not attend BCS as 1 (positive) and women who attended BCS as 0 (negative), we are interested in maximising the sensitivity (detection of true positives) and the balanced accuracy (average of sensitivity and specificity) and reducing the classification error. Moreover, the output of the decision trees (leaves) should have a reasonable size to use later as exposure variables for the logistic regression.

Considering this requirement, we decided to use the tune function for optimising the sensitivity (i.e. enhancing detection of positive cases) and the Area Under the Precision-Recall Curve (i.e. improving overall precision-recall performance for unbalanced datasets). **Table A** presents the chosen performance measures to evaluate the three compared decision trees and their values.

**Table A.** Performance measures of the three decision trees evaluated in the first step of analytical strategy b

| **Type of decision tree** | **Sensitivity** | **Specificity** | **NPV** | **PPV** | **ce** | **Balanced accuracy** | **Confusion matrix** |
| --- | --- | --- | --- | --- | --- | --- | --- |
| **CIT** | 0.7105263 | 0.4942583 | 0.9365009 | 0.1398964 | 0.4833018 | 0.6023923 | truth  response  1 0  1 351 2158  0 143 2109 |
| **CART** | 0.7246964 | 0.5134755 | 0.9415557 | 0.1470830 | 0.4646083 | 0.6190859 | truth  response  1 0  1 358 2076  0 136 2191 |
| **C50** | 0.5060729 | 0.6442465 | 0.9184764 | 0.1414027 | 0.3700903 | 0.5751597 | truth  response 1 0  1 250 1518  0 244 2749 |

| **Used code in R 4.4.0**  #load pacakges  Library(tidyverse)  library(mlr3verse)  library(mlr3learners)  library(mlr3tuningspaces)  library(mlr3tuning)  library(mlr3extralearners)  library(mlr3viz)  library(partykit)  library(rpart)  library(C50)  library(ggparty)  # Creating a classification task from our data  df <-  df %>%  mutate(  weights = ifelse(nevermam == 1, 7.588235, 1) )  mam_task <- TaskClassif$new(  id = "mam",  backend = df,  target = "nevermam",  positive="1")  mam_task$col_roles$weight="weights"  mam_task$set_col_roles(cols = c("weights"), remove_from = "feature")  #stratified sampling - setting stratified division in CV=5 (for imbalanced dataset)  rsmp_c5 = rsmp("cv", folds = 5)  mam_task$set_col_roles("nevermam", c("target", "stratum"))  rsmp_c5$instantiate(mam_task)  fold1 = prop.table(table(mam_task$data(rows = rsmp_c5$test_set(1), cols = "nevermam")))  fold2 = prop.table(table(mam_task$data(rows = rsmp_c5$test_set(2), cols = "nevermam")))  fold3 = prop.table(table(mam_task$data(rows = rsmp_c5$test_set(3), cols = "nevermam")))  fold4 = prop.table(table(mam_task$data(rows = rsmp_c5$test_set(4), cols = "nevermam")))  fold5 = prop.table(table(mam_task$data(rows = rsmp_c5$test_set(5),  cols = "nevermam")))  rbind("Fold 1" = fold1, "Fold 2" = fold2, "Fold 3"=fold3, "Fold 4"=fold4, "Fold 5"=fold5)  CIT  lrn_ctree = lrn("classif.ctree",  maxdepth= to_tune(1,7,logscale = F),  mincriterion = to_tune(0.01,0.99, logscale = F),  predict_type = "prob")  measures = msrs(c("classif.prauc", "classif.sensitivity"))  tuner=tnr("random_search")  instance = tune(  tuner=tnr("random_search"),  task = mam_task,  learner = lrn_ctree,  resampling = rsmp_c5,  measures = measures,  terminator = trm("evals", n_evals = 300),  store_models=T  )  tuner$optimize(instance)  > #inspect the best-performing configurations  instance$archive$best()[, .(mincriterion, maxdepth, classif.prauc, classif.sensitivity)]  mincriterion maxdepth classif.prauc classif.sensitivity  <num> <int> <num> <num>  1: 0.91012928 3 0.1224551 0.6719439  2: 0.42821453 3 0.1224551 0.6719439  3: 0.78950642 4 0.1291099 0.6195011  ctreeinst = lrn_ctree  > ctreeinst$param_set$values = instance$result_learner_param_vals[[1]]  >  > ctreeinst$train(mam_task)  > ctreeinst$model  Model formula:  nevermam ~ availablehelp + citizenship + cohabitation + education2 +  gali + incomehh + land + legalms + origin + perceivedinterest +  socialnetwork + typehh + urb + working  Fitted party:  [1] root  \| [2] typehh in 1, 2, 4, 5  \| \| [3] legalms in 1, 2, 4  \| \| \| [4] perceivedinterest in 1, 3, 4: 1 (w = 2011.8, err = 36.0%)  \| \| \| [5] perceivedinterest in 2, 5: 1 (w = 2211.1, err = 46.4%)  \| \| [6] legalms in 3  \| \| \| [7] incomehh in 1, 2, 3, 4: 0 (w = 629.5, err = 48.1%)  \| \| \| [8] incomehh in 5: 0 (w = 97.6, err = 9.2%)  \| [9] typehh in 3  \| \| [10] working in 1, 2, 5, 6  \| \| \| [11] land in 1, 4, 7, 8, 9, 10, 11, 13, 14, 15: 0 (w = 1430.1, err = 40.5%)  \| \| \| [12] land in 2, 3, 5, 6, 12, 16: 1 (w = 968.8, err = 42.0%)  \| \| [13] working in 3, 4  \| \| \| [14] land in 1, 4, 5, 8, 13, 15, 16: 0 (w = 258.6, err = 3.5%)  \| \| \| [15] land in 2, 3, 6, 7, 9, 10, 11, 12, 14: 0 (w = 929.1, err = 36.3%)  Number of inner nodes: 7  Number of terminal nodes: 8  > ctreeinstpred <- ctreeinst$predict(mam_task)  > ctreeinstpred$score(msrs(c("classif.acc", "classif.ce", "classif.npv", "classif.ppv", "classif.auc", "classif.bacc", "classif.fbeta", "classif.sensitivity", "classif.specificity")))  classif.acc classif.ce classif.npv  0.5166982 0.4833018 0.9365009  classif.ppv classif.auc classif.bacc  0.1398964 0.6368228 0.6023923  classif.fbeta classif.sensitivity classif.specificity  0.2337662 0.7105263 0.4942583  > ctreeinstpred$confusion  truth  response 1 0  1 351 2158  0 143 2109    > CTree <- ctree (nevermam ~ availablehelp + citizenship + cohabitation + education2 + gali + incomehh + land + legalms + origin + perceivedinterest + socialnetwork + typehh + urb + working,  + data=df_titanic_drop,  + weights=df_titanic_drop$weights,  + control = ctree_control(testtype="Bonferroni", mincriterion= 0.91012928, maxdepth=3))  > plot(CTree)    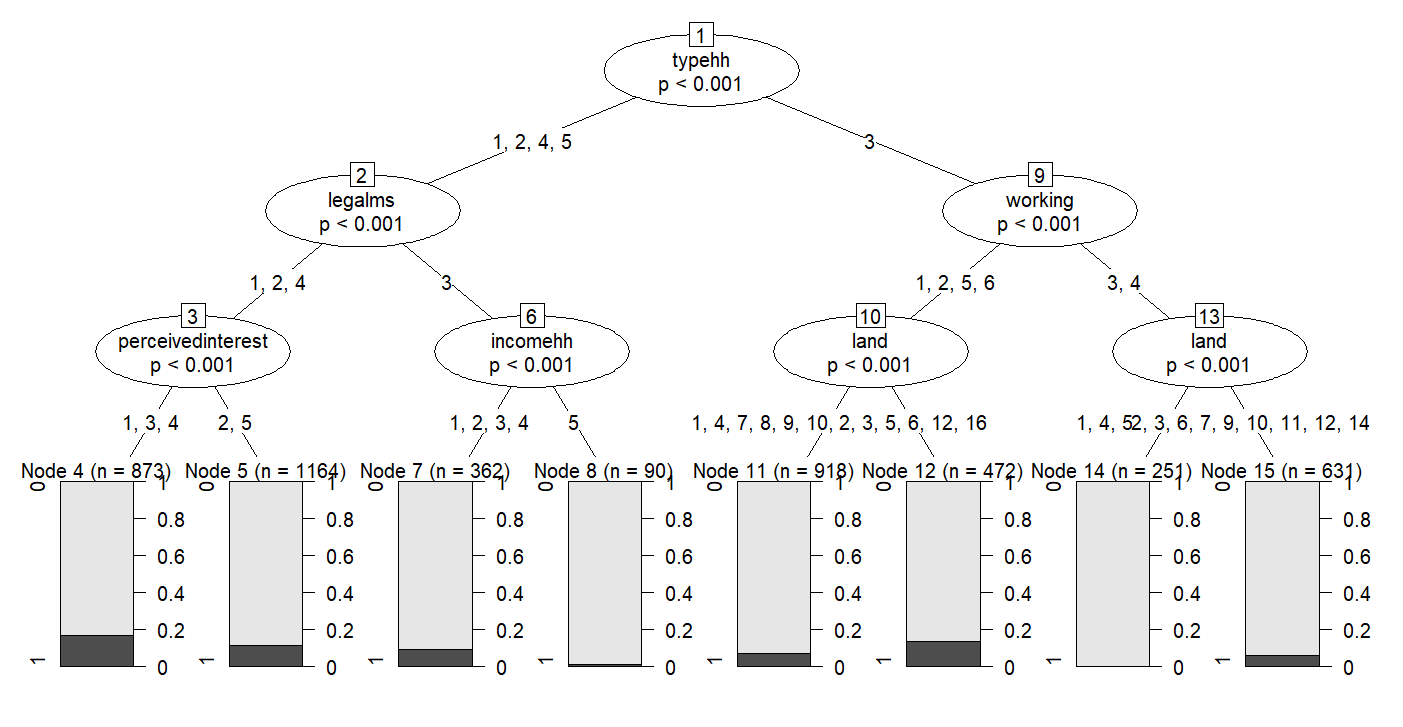  CART  lrn_cart = lrn("classif.rpart",  cp=to_tune(1e-04, 1e-1, logscale = F),  maxdepth=to_tune(1,4),  predict_type = "prob")  measures = msrs(c("classif.prauc", "classif. sensitivity"))  tuner=tnr("random_search")  instance = tune(  tuner=tnr("random_search"),  task = mam_task,  learner = lrn_cart,  resampling = rsmp_c5,  measures = measures,  terminator = trm("evals", n_evals = 300),  store_models=T  )  tuner$optimize(instance)  > instance$archive$best()[, .(cp, maxdepth, classif.prauc, classif.sensitivity)]  cp maxdepth classif.prauc classif.sensitivity  <num> <int> <num> <num>  1: 0.007483921 7 0.1352798 0.6317048  2: 0.008621253 5 0.1300750 0.6397856  3: 0.006117899 5 0.1358377 0.6115028  cartinst = lrn("classif.rpart")  cartinst$param_set$values = instance$result_learner_param_vals[[1]]  cartinst$train(mam_task)  cartinst$model  n= 4761  node), split, n, loss, yval, (yprob)  * denotes terminal node  1) root 4761 4267.0000 1 (0.5001587 0.4998413)  2) typehh=1,2,4,5 2489 2167.0000 1 (0.5622302 0.4377698)  4) legalms=1,2,4 2037 1751.0000 1 (0.5853597 0.4146403)  8) perceivedinterest=1,3,4 873 724.0000 1 (0.6401282 0.3598718) *  9) perceivedinterest=2,5 1164 1027.0000 1 (0.5355274 0.4644726)  18) typehh=1,2,4 953 829.0000 1 (0.5638561 0.4361439) *  19) typehh=5 211 112.3608 0 (0.3620328 0.6379672) *  5) legalms=3 452 311.1529 0 (0.4279058 0.5720942)  10) land=2,4,5,6,12 136 116.0000 1 (0.5984252 0.4015748) *  11) land=1,3,7,8,9,10,11,13,14,15,16 316 138.2902 0 (0.3155220 0.6844780) *  3) typehh=3 2272 1486.6200 0 (0.4144905 0.5855095)  6) working=1,2,5,6 1390 1140.8940 0 (0.4755917 0.5244083)  12) land=2,3,5,6,12,16 472 407.0000 1 (0.5798943 0.4201057) *  13) land=1,4,7,8,9,10,11,13,14,15 918 579.0902 0 (0.4049326 0.5950674) *  7) working=3,4 882 345.7255 0 (0.2910820 0.7089180) *  ) *  cartinst_pred <- cartinst$predict(mam_task)  cartinst_pred$score(msrs(c("classif.acc", "classif.ce","classif.npv", "classif.ppv", "classif.auc", "classif.bacc", "classif.fbeta", "classif.sensitivity", "classif.specificity")))  classif.acc classif.ce classif.npv classif.ppv  0.5353917 0.4646083 0.9415557 0.1470830  classif.auc classif.bacc classif.fbeta classif.sensitivity  0.6410595 0.6190859 0.2445355 0.7246964  classif.specificity  0.5134755  > tuned_cart_pred$confusion  truth  response 1 0  1 358 2076  0 136 2191  > learner = lrn("classif.rpart", keep_model = TRUE, cp=0.01420474, maxdepth=4, predict_type = "prob")  > learner$train(mam_task)  > pred = learner$train(mam_task)$predict(mam_task)  > autoplot(learner,type = "ggparty")  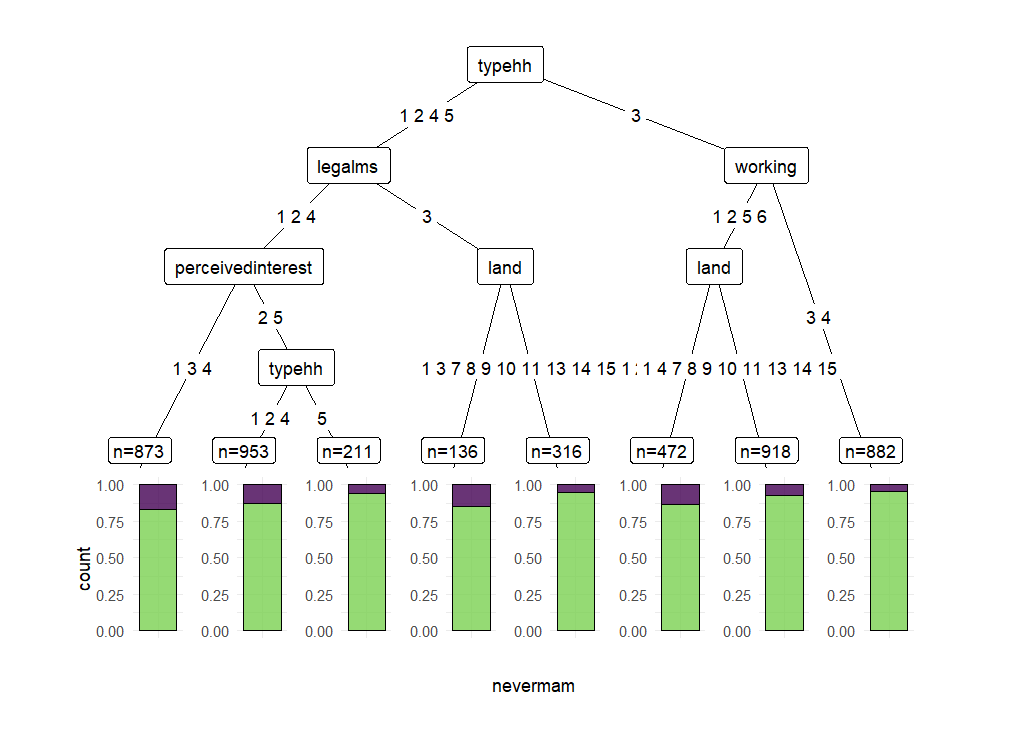  C50  lrn_c50 = lrn("classif.C50",  winnow=T,  CF=to_tune(0,1),  minCases=to_tune(240,330),  predict_type = "prob")  measures = msrs(c("classif.prauc", "classif.sensitivity"))  tuner=tnr("random_search")  instance = tune(  tuner=tnr("random_search"),  task = mam_task,  learner = lrn_c50,  resampling = rsmp_c5,  measures = measures,  terminator = trm("evals", n_evals = 300),  store_models=T  )  instance  tuner$optimize(instance)  instance$archive$best()[, .(CF, minCases, classif.prauc, classif.sensitivity)]  CF minCases classif.prauc classif.sensitivity  <num> <int> <num> <num>  1: 0.476114000 241 0.1300718 0.6515564  2: 0.346356111 316 0.1218811 0.6356834  3: 0.218427168 252 0.1267659 0.6071119   \| > c50inst$train(mam_task)  > c50inst$tuning_result  > c50inst_pred <- c50inst$predict(mam_task)  > c50inst_pred$score(msrs(c("classif.acc", "classif.ce","classif.npv", "classif.ppv", "classif.auc", "classif.bacc", "classif.fbeta", "classif.sensitivity", "classif.specificity")))  classif.acc classif.ce classif.npv classif.ppv  0.6299097 0.3700903 0.9184764 0.1414027  classif.auc classif.bacc classif.fbeta classif.sensitivity  NaN 0.5751597 0.2210433 0.5060729  classif.specificity  0.6442465  > c50inst_pred$confusion  truth  response 1 0  1 250 1518  0 244 2749  > set.seed(123456)  > c5cntrol<-C5.0Control(winnow=T,  + CF= 0.476114000,  + minCases=241,  + earlyStopping=F)  >  >  > c50.1 <- C5.0(nevermam ~ availablehelp + citizenship + cohabitation + education2 + gali + incomehh + land + legalms + origin + perceivedinterest + socialnetwork + typehh + urb + working,  + data = df_titanic_drop,  + weights=df_titanic_drop$weights,  + trials=1,  + control=c5cntrol)  > plot(c50.1) \| \| --- \|   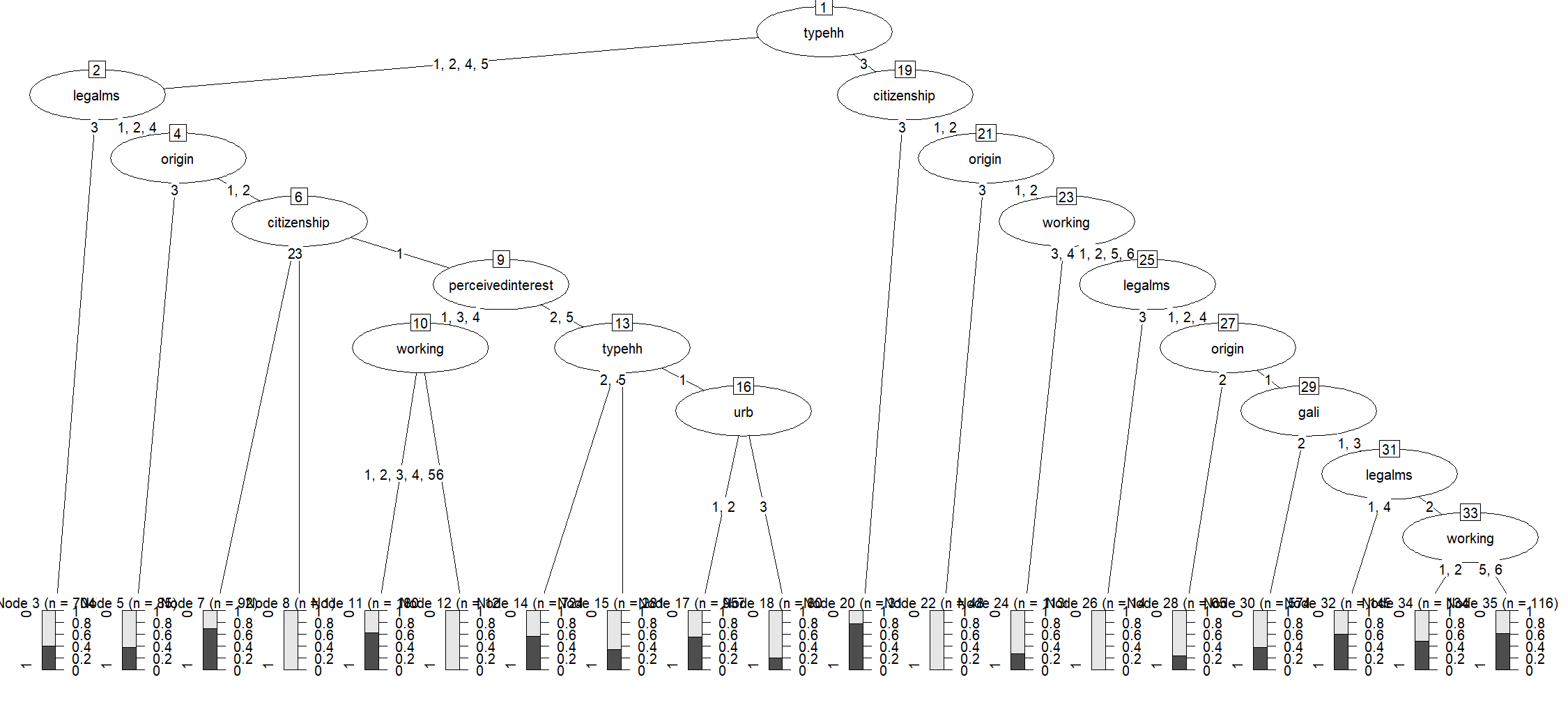 |
| --- | --- |
